# Combined Use of Autofluorescence and Indocyanine Green Fluorescence Imaging in the Identification and Evaluation of Parathyroid Glands During Total Thyroidectomy: A Randomized Controlled Trial

**DOI:** 10.3389/fendo.2022.897797

**Published:** 2022-06-16

**Authors:** Supeng Yin, Bin Pan, Zeyu Yang, Mi Tang, Hongbiao Mo, Yao Li, Ziying Yi, Tingjie Yin, Cong Shao, Cunye Yan, Linlong Mo, Yuquan Yuan, Yiceng Sun, Fan Zhang

**Affiliations:** ^1^ Department of Breast and Thyroid Surgery, Chongqing General Hospital, Chongqing, China; ^2^ Graduate School of Medicine, Chongqing Medical University, Chongqing, China

**Keywords:** parathyroid glands, near-infrared fluorescence imaging, autofluorescence, indocyanine green, thyroidectomy

## Abstract

**Background and objectives:**

Accurate identification and evaluation of the parathyroid glands (PGs) intraoperatively is critical to reduce the incidence of postoperative hypoparathyroidism after total thyroidectomy. Near-infrared fluorescence imaging (NIFI), including the autofluorescence (AF) and indocyanine green fluorescence (ICGF) imaging, is a promising technique to protect PGs. This study aimed to assess whether the combined use of AF and ICGF could reduce the incidence of postoperative hypoparathyroidism and improve the identification and evaluation of PGs during total thyroidectomy.

**Methods:**

This randomized controlled trial enrolled 180 patients who were randomized into two groups and underwent total thyroidectomy with unilateral or bilateral central lymph node dissection. In the control group, the PGs were identified and evaluated by the naked eye. In the NIFI group, AF was used to identify the PGs and ICGF was applied to assess the blood perfusion of the PGs *in situ*. The primary outcome was the incidence of postoperative hypoparathyroidism. The secondary outcomes included the number of identified PGs, autotransplanted PGs, and known preserved PGs *in situ*.

**Results:**

The incidence of postoperative transient hypoparathyroidism was significantly lower in the NIFI group than in the control group (27.8% vs. 43.3%, *P* = 0.029). More PGs were identified in the NIFI group than in the control group (3.6 ± 0.5 vs. 3.2 ± 0.4, *P* < 0.001). No significant difference was observed in the number of autotransplanted PGs between the two groups (*P* = 0.134). Compared with the control group, a greater number of known PGs were preserved *in situ* in the NIFI group (1.3 ± 0.6 vs. 1.0 ± 0.5, *P* < 0.001). In the NIFI group, only 4.5% of the patients with at least one well-perfused PG (ICG score of 2) developed postoperative hypoparathyroidism, which was significantly lower than that of the control group (34.6%, *P* < 0.001).

**Conclusion:**

Combined use of AF and ICGF during total thyroidectomy reduces the risk of transient postoperative hypoparathyroidism, enhances the ability to identify and preserve PGs, and improves the accuracy of evaluating the perfusion of PGs during surgery.

**Clinical Trial Registration:**

Chinese Clinical Trial Register (www.chictr.org.cn), identifier ChiCTR2100045320. Registered on April 12, 2021.

## Introduction

Postoperative hypoparathyroidism or hypocalcemia is the most frequent complication after total thyroidectomy (TT) (1–3). The median incidence of transient and permanent postoperative hypoparathyroidism is 27% (19%-38%) and 1% (0-3%), respectively (4). Transient hypoparathyroidism can increase the length of hospital stay and the costs of hospitalization for patients. Permanent hypoparathyroidism leads to lifelong medication use, which may severely impair the quality of life of patients. Therefore, identifying the parathyroid glands (PGs) and preserving those with adequate blood perfusion intraoperatively has always been a crucial procedure during thyroidectomy.

Conventional means of identification and assessment of PGs are mainly based on surgeon-dependent identification of their anatomical location and appearance (color, shape, etc.) by the naked eye. However, this visual inspection is often influenced by the experience of the surgeon, intraoperative hemorrhage, ectopic PGs and so on, which makes it difficult to fully protect the PGs.

Currently, there has been an emergence of near-infrared fluorescence imaging (NIFI) that can be applied during thyroid or parathyroid surgery for the evaluation and identification of PGs (5–8). This technique mainly exploits the autofluorescence (AF) of PGs and indocyanine green fluorescence (ICGF) imaging. AF of PGs was first discovered by Das et al. in 2006 (9). In 2011, Paras et al. used near-infrared AF imaging to identify PGs in surgery (10). They discovered that PGs could exhibit stronger AF than the surrounding tissue under near-infrared light, which can be exploited to accurately identify PGs in real time. Subsequent studies have demonstrated that AF can not only detect PGs intraoperatively in real time and improve the intraoperative identification of PGs but also reduce the incidence of postoperative hypoparathyroidism (11–14). However, AF cannot assess the status of PG blood perfusion and consequently is not useful when determining whether PGs need to be autotransplanted (8).

Indocyanine green (ICG) fluorescence imaging may be a great solution to this problem. ICG, first approved for clinical use in 1956, is a safe fluorescent dye with fast metabolism and few adverse effects that can quickly combine with plasma proteins after intravenous injection and is widely used in angiography in multiple surgical disciplines (15, 16). In recent years, several studies have shown that ICGF may be superior in evaluating blood perfusion and predicting the function of PGs *in situ*, subsequently guiding their autotransplantation (17–20). However, ICGF may not be very suitable for the identification of PGs before dissection, as the thyroid gland would also emit intense fluorescence after the injection of ICG, which may lead to difficulties in distinguishing them (17). Thus, AF and ICGF have their own advantages and disadvantages in the identification and evaluation of PGs, respectively. These two methods can be considered complementary.

In the present study, we used AF in combination with ICGF imaging by one fluorescence imaging system during different steps of the surgical procedure. At the beginning of the operation, AF was used to identify the PGs before any dissection. Then, after the thyroid gland was removed, AF was used again to locate the PGs *in situ*. Finally, ICGF was applied to evaluate the blood perfusion of the PGs *in situ* and guide their autotransplantation. The aim of this randomized controlled trial was to assess whether this strategy could reduce the incidence of postoperative hypoparathyroidism and benefit the identification and evaluation of PGs during total thyroidectomy.

## Materials And Methods

### Study Design

**Figure 1:**
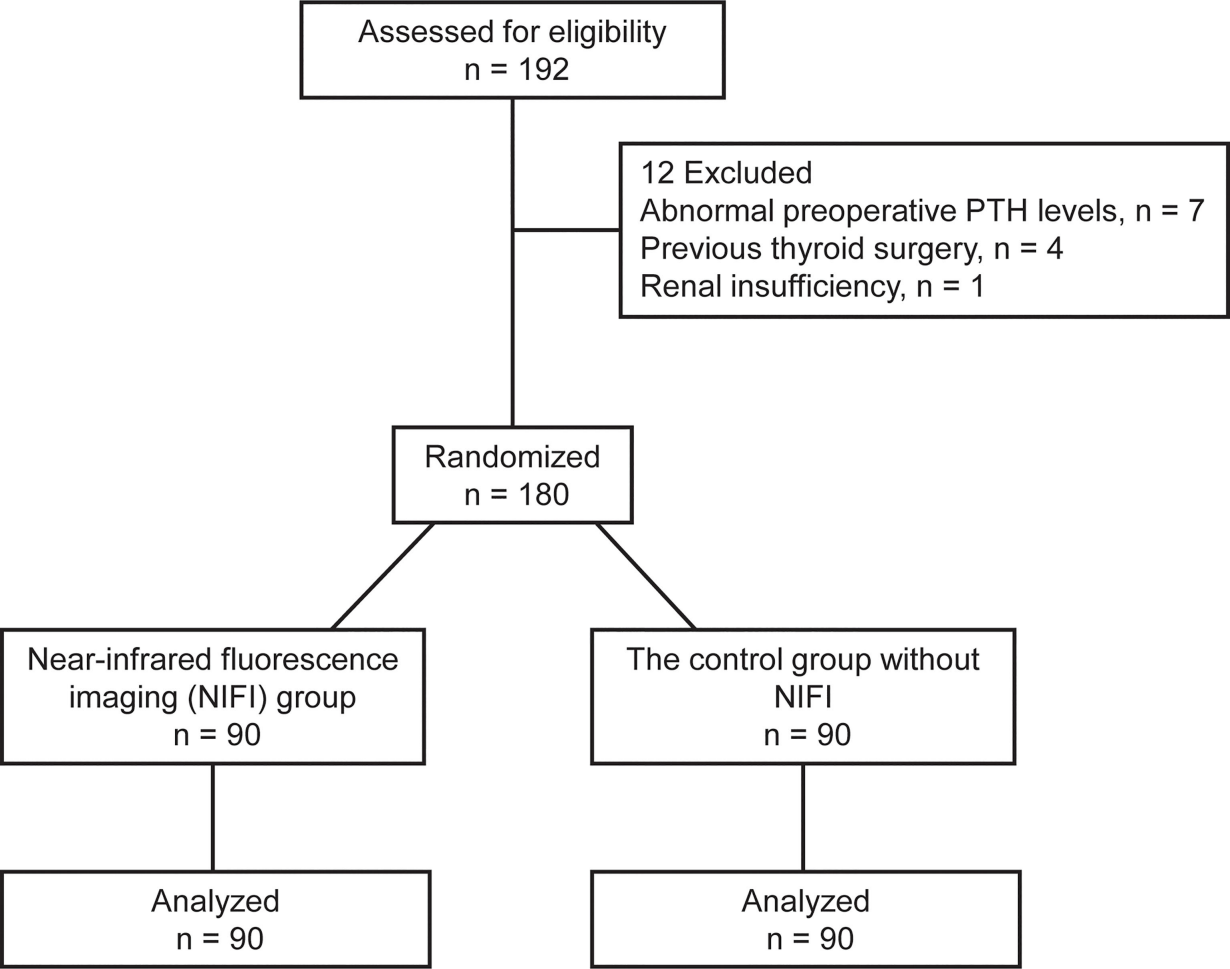
Study flowchart.

This randomized controlled trial was conducted in the Department of Breast and Thyroid Surgery of Chongqing General Hospital from May 2021 to January 2022. Patients between 18 and 60 years old who were diagnosed with papillary thyroid cancer by fine needle aspiration biopsy with indications for total thyroidectomy were eligible for the study. Those with abnormal preoperative parathyroid hormone (PTH) levels or comorbid parathyroid disease, hepatic or renal insufficiency, previous thyroid or parathyroid gland surgery, or allergy to ICG were excluded. Patients were randomly assigned to the NIFI group, which received the fluorescence imaging technique, or the control group without NIFI in a 1:1 ratio using a computer-generated randomization scheme (**Figure 1**).

This study was approved by the Ethics Review Board of Chongqing General Hospital and registered in the Chinese Clinical Trial Registry (ChiCTR2100045320). Written informed consent was obtained from all participants.

### Imaging System

A real-time image-guided system (Nanjing Nuoyuan Medical Devices Co., Ltd. Nanjing, China) was used in this study, which consists of a handheld laser probe, a camera and a visualization system with two light sources. Light source I (excitation light 785 nm, reception light 835 nm) is a point light source that can stimulate the autofluorescence of PGs and requires the operator to hold the probe to irradiate the parathyroid gland. Light source II (excitation light 785 nm, reception light 815 nm) is a surface light source, which is a part of the ICG fluorescence system and does not require the operator to hold the probe. When used in this surgery, the camera is covered by a dedicated sterile cover and placed 300-330 mm above the surgical field. Subsequently, the lights of the operating room are turned off, and the different light sources are switched on as needed for different types of fluorescence imaging.

### Surgical Procedures

All operations were performed by one experienced endocrine surgeon. A transverse incision was made, flap dissection was conducted, and the anterior cervical musculature was retracted laterally to expose the thyroid. The upper pole of the thyroid gland was severed on one side, and then the thyroid gland was turned to the opposite side to expose the back of the thyroid gland.

**Figure 2:**
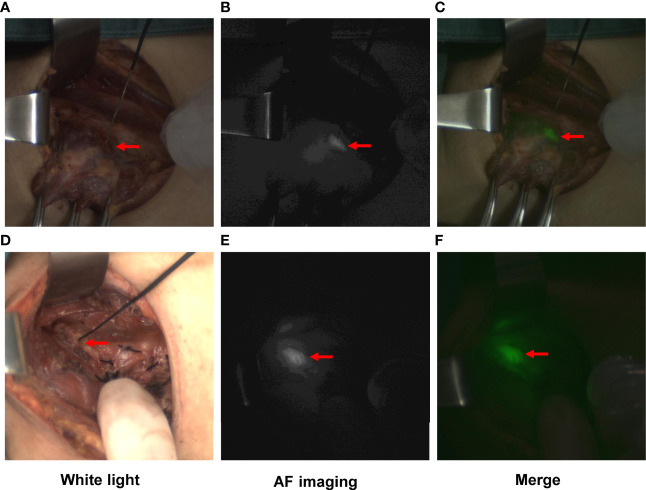
Typical images of PGs visualized by autofluorescence (AF) imaging. PGs exposed to white light **(A, D)**, near-infrared light **(B, E)**, and the merged images **(C, F)**. Images **(A–C)** show the PG identified before any dissection. Images **(D–F)** show the PG *in situ* detected by AF after complete removal of the thyroid gland.

Then, the PGs were identified by the naked eye or NIFI before any dissection. The camera was moved 300-330 mm above the surgical field in a sterile sleeve, and the fluorescence system was set to Light Source I. The operating room lights were turned off, and the laser probe was held to illuminate the back of the thyroid gland to find the PGs *in situ* with autofluorescence (**Figure 2**). Then, the operation was continued, and the PGs were separated and protected carefully. The procedure for the opposite lobe was the same.

**Figure 3:**
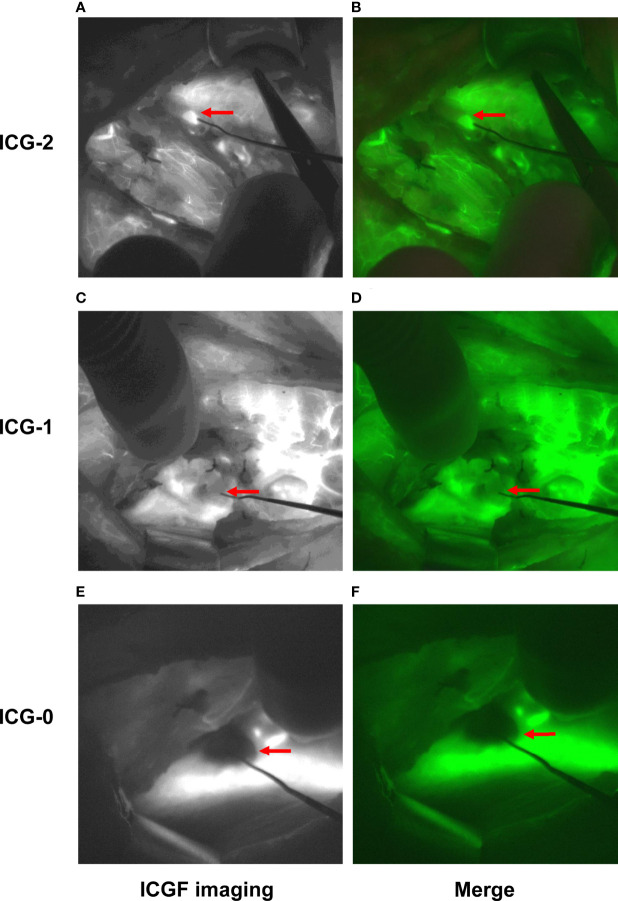
Typical images of PGs assessed by ICGF. Fluorescence images were obtained 30 seconds after intravenous injection of ICG. Images **(A, B)** show the PG with an ICG score of 2. Images **(C, D)** show the PG with an ICG score of 1. Images **(E, F)** show the PG with an ICG score of 0.

After complete removal of the thyroid gland, in the control group, PGs *in situ* were detected, and their blood perfusion was evaluated by the naked eye following the principle given in **Table 1**. In the NIFI group, the camera was moved back, and the PGs *in situ* were detected by AF again (**Figure 2**). Then, the fluorescence system was set to light source II, and 2.5 mg of ICG was injected intravenously to obtain fluorescence images after 30 seconds. The perfusion of the PGs was then evaluated as described by Fortuny et al. (21) (**Table 1** and **Figure 3**). The PGs with ICG or visual score of 0 and the PGs found in the resected specimens in both groups were autotransplanted into the sternocleidomastoid muscle.

**Table 1:** Intraoperative parathyroid gland (PG) scoring.

Visual score	Description
Score 0	No blood perfusion
Score 2	Good blood perfusion
ICG score	
Score 0	Black PG (no blood perfusion)
Score 1	Gray PG (partial blood perfusion)
Score 2	White PG (good blood perfusion)

### Outcomes

The primary outcome was postoperative hypoparathyroidism, which was defined as a PTH level lower than 12 pg/mL postoperatively. Both the PTH levels (reference range 12-88 pg/mL, Beckman Coulter Unicel DxI 800) and the serum calcium levels were measured and recorded in all of the patients before surgery and on postoperative day one to assess the parathyroid function. The PTH levels of the patients with postoperative hypoparathyroidism were measured again at 3 days, 14 days, 1 month, 3 months, or 6 months postoperatively. If the PTH level increased to a normal level within 6 months postoperatively, it was defined as transient hypoparathyroidism; otherwise, it was defined as permanent hypoparathyroidism. Patients were also observed for symptoms of hypocalcemia. Patients with hypoparathyroidism were treated with oral calcium supplementation or intravenous calcium supplementation depending on their serum calcium levels and their symptoms.

Secondary outcomes included the number of PGs detected during the surgery, the number of autotransplanted PGs, and the number of known preserved PGs *in situ* (including those identified PGs with a visual score of 2 and an ICG score of 1 or 2). Finally, the incidence of hypoparathyroidism in patients with at least one well-perfused PG in both groups was compared.

### Statistical Analysis

The primary outcome, the incidence of transient hypoparathyroidism, was used to calculate the sample size. The incidence of transient hypoparathyroidism was assumed to be 45% according to some previous studies (19, 22, 23) and the data in our center. Assuming a 50% relative reduction in transient hypoparathyroidism, with 95% confidence (α = 0.05) and 90% power (β = 0.10), a sample size of 88 patients per group was estimated to be necessary. Taking into consideration the potential subject loss, we finally included 90 participants in each group.

Continuous data were recorded as the mean and standard deviation. Categorical data were recorded as counts and percentages. The incidence of postoperative hypoparathyroidism, the number of PGs detected during the surgery, the number of autotransplanted PGs, and the number of known preserved PGs *in situ* were compared between the two groups. Finally, the incidence of hypoparathyroidism in patients with at least one well-perfused PG in both groups was analyzed. Continuous variables were analyzed using nonpaired Student’s *t-test*, and categorical variables were analyzed using Pearson’s chi-square analysis. All analyses were bilateral and assessed using a significance criterion of α = 0.05 and conducted using SPSS (version 26.0, SPSS Inc.) by a professional statistician.

## Results

A total of 180 patients were enrolled in the present study: 90 patients in the NIFI group using AF in combination with ICGF during the operation and 90 in the control group without NIFI. All of the patients were diagnosed with papillary thyroid cancer and underwent total thyroidectomy with unilateral or bilateral central lymph node dissection (CLND). In the NIFI group, unilateral CLND was performed in 32.2% of patients, and bilateral CLND was performed in 67.8% of patients. In the control group, these ratios were 36.7% and 63.3%, respectively. There was no significant difference between the groups in terms of CLND (*P* = 0.530). In addition, as summarized in **Table 2**, the two groups were comparable in terms of all baseline characteristics.

**Table 2:** Demographics and baseline characteristics of the participants.

	NIFI group (n=90)	Control group (n=90)	*χ* ^2^(*t*) Value	*P* Value
Age, mean ± SD	42.1 ± 9.5	42.7 ± 9.1	0.369	0.713
Sex, n (%)			0.556	0.456
Male	20 (22.2%)	16 (17.8%)		
Female	70 (77.8%)	74 (82.2%)		
BMI, mean ± SD	23.6 ± 3.0	23.5 ± 2.7	-0.244	0.807
Size of the largest tumor, mean ± SD, mm	14.3 ± 8.5	13.2 ± 8.1	-0.934	0.351
Preoperative PTH, mean ± SD (pg/mL)	49.9 ± 15.5	48.1 ± 14.1	-0.835	0.405
CLND, n (%)			0.394	0.530
Unilateral	29 (32.2%)	33 (36.7%)		
Bilateral	61 (67.8%)	57 (63.3%)		
No. of resected LNs, mean ± SD	11.0 ± 6.7	9.9 ± 6.4	-1.097	0.274
No. of metastatic LNs, mean ± SD	2.8 ± 4.2	2.1 ± 2.6	-1.363	0.175

BMI, body mass index; PTH, parathyroid hormone; CLND, central lymph node dissection; LNs, lymph nodes.

The serum calcium levels and PTH levels of all patients were detected on postoperative day one. No significant difference was observed in the serum calcium levels between the groups (2.2 ± 0.1 mmol/L in the NIFI group vs. 2.1 ± 0.2 mmol/L in the control group, *P* = 0.051). As the change in postoperative serum calcium levels is often lagging and may be influenced by calcium supplementation in different patients, we evaluated the parathyroid function based on PTH levels. The PTH levels of the patients in the NIFI group on postoperative day one were significantly higher than in the control group (21.1 ± 16.0 pg/mL vs. 15.0 ± 12.9 pg/mL, *P* < 0.01). As shown in **Table 3**, the primary outcome, the incidence of postoperative transient hypoparathyroidism, was significantly lower in the NIFI group than in the control group (27.8% vs. 43.3%, *P* = 0.029). No patients developed permanent hypoparathyroidism in either group, and the PTH levels of all patients who suffered transient hypoparathyroidism increased to the normal reference range during the follow-up period.

**Table 3:** Outcomes.

	NIFI group (n=90)	Control group (n=90)	*χ* ^2^ (*t*) Value	*P* Value
Primary outcome				
Transient hypoparathyroidism, n (%)	25 (27.8%)	39 (43.3%)	4.752	0.029*
Permanent hypoparathyroidism, n (%)	0	0		
Secondary outcomes				
No. of identified PGs, mean ± SD	3.6 ± 0.5	3.2 ± 0.4	-6.017	<0.001*
No. of autotransplanted PGs, mean ± SD	2.3 ± 0.6	2.2 ± 0.4	-1.505	0.134
No. of known preserved PGs *in situ*, mean ± SD	1.3 ± 0.6	1.0 ± 0.5	-3.713	<0.001*

*Significant; PG, parathyroid gland.

Analysis of the number of PGs detected during the surgery showed that there were more PGs identified in the NIFI group than in the control group (*P* < 0.001), with a mean of 3.6 (SD 0.5) PGs found in the NIFI group and 3.2 (SD 0.4) PGs in the control group (**Table 3**), indicating that AF significantly improves the ability to identify PGs during surgery.

The average number of autotransplanted PGs was 2.3 (SD 0.6) in the NIFI group and 2.2 (SD 0.4) in the control group. No significant difference was observed between the groups (**Table 3**). For the number of known PGs ultimately preserved *in situ*, a significant difference was observed between the NIFI group and the control group (1.3 ± 0.6 vs. 1.0 ± 0.5, *P* < 0.001) (**Table 3**).

In the NIFI group, 66 patients had at least one well-perfused PG (ICG score of 2), and only 4.5% of these patients developed hypoparathyroidism at postoperative day one. In the control group, 78 patients were evaluated as having at least one well-perfused PG by the naked eye, but 34.6% of those patients developed postoperative hypoparathyroidism, which was significantly higher than that of the NIFI group (*P* < 0.001) (**Table 4**). This result suggested that ICGF assessment of PG vascularity was more accurate than visual assessment.

**Table 4:** The incidence of hypoparathyroidism in patients with at least one well perfused (score 2) PG.

	NIFI group (n=66)	Control group (n=78)	*χ*^2^ (*t*) Value	*P* Value
Transient hypoparathyroidism	3 (4.5%)	27 (34.6%)	19.599	<0.001*
Normal postoperative PTH	63 (95.5%)	51 (65.4%)		

*Significant; PG, parathyroid gland; PTH, parathyroid hormone.

## Discussion

Hypoparathyroidism is the most frequent complication after total thyroidectomy. Although great attention has been given to the identification and preservation of PGs, inadvertent excision or devascularization cannot be completely avoided, even by experienced surgeons. In recent years, the technique of near-infrared fluorescence imaging (NIFI), which includes the autofluorescence (AF) of PGs and indocyanine green fluorescence (ICGF) imaging, seems to be a promising method to protect PGs in thyroid surgery. However, both methods have their own advantages and disadvantages. AF will help to locate the PGs but cannot provide information about the viability of the glands (8, 13). ICGF imaging has the advantage of assessing the viability of the glands by evaluating the vascular supply (17, 18).

Many studies have compared AF or ICGF imaging with the conventional naked eye for PG detection and evaluation (11, 12, 19, 21) but have rarely considered combining these two methods in one operation. In our opinion, these two techniques are complementary. Their combined use may achieve the protection of PGs throughout the whole procedure during thyroidectomy. Alesina et al. first showed the feasibility of this strategy during video-assisted neck surgery but did not demonstrate any superiority, such as reducing the incidence of postoperative hypoparathyroidism or improving the ability of PG identification (24).

In the present study, we used AF imaging to identify and locate PGs before any dissection of the thyroid in the first step. Then, after the thyroid gland was removed, ICGF imaging was applied to assess the perfusion of the PGs and guide their autotransplantation. Our data showed that the combined use of AF and ICGF during total thyroidectomy significantly reduced the incidence of transient postoperative hypoparathyroidism and improve the identification of PGs compared with conventional naked eye surgery.

In previous studies, AF was demonstrated to be an effective way to detect PGs. Our data also showed that the average number of PGs per patient detected by AF was greater than that after conventional naked eye surgery, which is consistent with the reports of Falco et al. and Benmiloud et al. (11, 13). Moreover, in the NIFI group, the number of known PGs preserved *in situ* was greater than that in the control group. These data demonstrated that this technique improves the ability to identify PGs and increases the possibility of preserving the glands *in situ*, which may be the primary cause of the decreased incidence of transient postoperative hypoparathyroidism.

Moreover, we observed that ICGF assessment of gland vascularity was more accurate than visual assessment. At least one PG with good perfusion (defined as an ICG score of 2) predicted an extremely high rate of normal PTH levels in the patients postoperatively. This result was concordant with the observation of Fortuny et al. (21). However, Rudin et al. reported that at least two vascularized glands detected on ICGF were required for normal parathyroid function (19). Meanwhile, they suggested that as the accuracy and sensitivity were only 63% and 72%, respectively, ICGF could not be used as the sole method for predicting postoperative hypoparathyroidism. This discrepancy may be attributed to the subjectivity of evaluating the ICG score. Quantitative evaluation of the fluorescence intensity may be a solution and will provide a more accurate and objective assessment. Lang et al. demonstrated that the index of the greatest and average fluorescent light intensity (FI) could quantitatively evaluate the perfusion of PGs and predict the postoperative hypocalcemia risk (25). Overall, we thought that the ability of ICGF to evaluate the perfusion of PGs is reliable enough and could guide the autotransplantation of PGs accurately.

The influence of these two techniques on PG autotransplantation is still uncertain. Rudin et al. found that the frequency of autotransplantation increased significantly when using ICGF to evaluate the perfusion of PGs in comparison to the non-ICGF group (19). They concluded that ICGF could guide more appropriate autotransplantation. A multicenter randomized clinical study suggested that fewer patients experienced autotransplantation in the AF group than in the control group without AF (13). They thought that AF could help surgeons preserve the PGs during total thyroidectomy. However, our results showed that there was no significant difference in the number of autotransplanted PGs between the two groups, which may be due to a combination of the two factors mentioned above. In addition, it is worth noting that our data showed a relatively higher rate of autotransplantation in both groups of patients than most previous studies. This is primarily because all the patients enrolled in this study underwent total thyroidectomy with unilateral or bilateral central lymph node dissection, so the higher number of devascularized or autotransplanted PGs is reasonable. In addition, we followed the consensus of the Chinese Thyroid Association on the protection of PGs and performed at least 1 PG autotransplantation in every total thyroidectomy. This strategy might also have contributed to the very low incidence of permanent postoperative hypoparathyroidism (approximately 0.42%) in our center.

However, despite these findings, there were several limitations to the current study. First, this study was conducted by one surgeon in a single institution. Moreover, as mentioned by other studies, the use of the imaging system may act primarily by reminding the surgeon to pay more attention to identifying and preserving the PGs. A multicenter study including more patients and surgeons is necessary. In addition, the large camera of the fluorescence imaging device used in our study needs to be improved because of its limitation in observing the PGs in some marginal or shaded areas. A smaller or handheld camera may solve this problem.

## Conclusion

In conclusion, the results of the current study suggest that the combined use of AF and ICGF during total thyroidectomy reduces the risk of transient postoperative hypoparathyroidism, enhances the ability to identify and preserve PGs, and improves the accuracy of evaluating the perfusion of PGs during the surgery. This strategy may help surgeons achieve full protection of PGs during thyroidectomy.

## Data Availability Statement

The datasets analyzed in the current study are available from the corresponding authors on reasonable request.

## Ethics Statement

The studies involving human participants were reviewed and approved by Ethics Review Board of Chongqing General Hospital. The patients/participants provided their written informed consent to participate in this study. Written informed consent was obtained from the individual(s) for the publication of any potentially identifiable images or data included in this article.

## Author Contributions

The authors have made the following declarations about their contributions: FZ, YS, and SY conceived and designed this study. SY, BP, YS, MT, HM, YL, ZiY, TY, CS, CY, LM, and YY performed the trial and collected the data; SY, ZeY, and BP analyzed the data. FZ, SY, and BP drafted the manuscript. All authors contributed to the article and approved the submitted version.

## Funding

This work was supported by the Chongqing Medical Scientific Research Project (Joint Project of Chongqing Health Commission and Science and Technology Bureau) (Grant No. 2021MSXM314) and the Medical Science and Technology Innovation Fund of Chongqing General Hospital (Grant No. 2019ZDXM01).

## Conflict of Interest

The authors declare that the research was conducted in the absence of any commercial or financial relationships that could be construed as a potential conflict of interest.

## Publisher’s Note

All claims expressed in this article are solely those of the authors and do not necessarily represent those of their affiliated organizations, or those of the publisher, the editors and the reviewers. Any product that may be evaluated in this article, or claim that may be made by its manufacturer, is not guaranteed or endorsed by the publisher.
